# The role of MTNR1B genetic polymorphisms in type 2 diabetes mellitus

**DOI:** 10.1186/1471-2164-15-S2-P1

**Published:** 2014-04-02

**Authors:** Nehad Shaer, Jalaluddin J Khan, Ashraf Dallol, Adel Abuzenadah

**Affiliations:** 1 KACST Technology Innovation Center in Personalized Medicine, King Abdulaziz University, KSA; 2 Department of Biochemistry, Faculty of Science, King Abdulaziz University, KSA

## Background

Type 2 diabetes (T2DM) is the most common form of diabetes and is characterized by obesity and physical inactivity. T2DM is a complex and pleomorphic metabolic disorder arising from a complex interaction between genes and the environment. However, the molecular landscape of T2DM is not fully explored, especially in a highly consanguineous society as the Saudi Arabian population [1]. As well as T2DM causes, genetics can affect response to treatment.

## Materials and methods

We have analyzed a cohort of 200 T2DM samples obtained from the city of Makkah for the presence of mutations or single nucleotide polymorphisms in the coding region of the melatonin receptor (MTNR1B; a major player in the regulation of the circadian clock) using PCR-sequencing method.

## Results

No mutations could be identified in the MTNR1B gene in our cohort. However, SNP rs60474139 was found at a frequency of 13.5%. The role of this SNP in maintaining Hb1Ac levels was investigated, however, no statistically significant associations could be found. Another SNP (rs10830962) in the MTNR1B introns previously reported to be associated with T2DM was investigated. However no significant association could be found with Hb1Ac levels.

## Conclusions

Although we could rule out a significant role of MTNR1B in T2DM in Saudi Arabia, we cannot dismiss the possibility of the existence of epigenetic mechanisms of inactivation. Further work is warranted in order to elucidate such mechanisms and explore the potential role of other circadian clock genes in the pathogenesis of T2DM.
